# Cause-specific hazards of antiretroviral therapy programmatic adherence, defaulting and mortality among HIV/AIDS patients who had attained CD4 count recovery after antiretroviral therapy initiation in South Africa

**DOI:** 10.3389/frph.2026.1797297

**Published:** 2026-05-12

**Authors:** Chiedza Elvina Mashiri, Knowledge Chinhamu, Jesca Mercy Batidzirai, Retius Chifurira

**Affiliations:** 1 Department of Applied Mathematics and Statistics, Midlands State University, Gweru, Zimbabwe; 2 School of Mathematics, Statistics and Computer Science, University of KwaZulu-Natal, Durban, South Africa; 3 School of Mathematics, Statistics and Computer Science, University of KwaZulu-Natal, Pietermaritzburg, South Africa

**Keywords:** adherence, cause-specific hazard, CD4 count recovery, competing risk, defaulting, mortality

## Abstract

**Background:**

Interruptions in treatment due to non-adherence or defaulting may compromise sustained immunological recovery among HIV patients who achieved viral suppression after antiretroviral therapy (ART) initiation. This study identified determinants of time to HIV-related mortality, ART defaulting, and adherence among PLWH who achieved CD4 count recovery.

**Methods:**

A retrospective cohort study analysed data from 726 HIV-positive patients, who achieved CD4 recovery between June 2004 and August 2013. Programmatic adherence, mortality, and defaulting were modelled as competing events to assess CD4 recovery trajectories, with outcomes mutually exclusive during follow-up. Programmatic adherence was defined as continuous engagement in care, measured by attending scheduled clinic visits and obtaining timely ART refills without interruptions of 90 or more consecutive days. The cumulative incidence function estimated probabilities and evaluated covariate effects, including age, sex, tuberculosis status, location, baseline CD4 count, and viral load. Bivariate cause-specific competing risk analyses were conducted for baseline characteristics, and variables with *p* < 0.20 were included in multivariable cause-specific hazard models.

**Results:**

Gender significantly predicted treatment default, with females having a higher hazard of default than males (aCSHR = 2.161; CI: 1.182–3.958). A higher baseline CD4 count was associated with an increased hazard of defaulting (aCSHR = 1.005; CI: 1.000–1.010). Age was the only significant predictor of mortality; increasing age was associated with greater mortality risk (aCSHR = 1.091; CI: 1.036–1.149). Females had a higher hazard of achieving adherence than males (aCSHR = 1.314; CI: 1.099–1.571). Patients without tuberculosis had a lower hazard of adherence than those with tuberculosis (aCSHR = 0.570; CI: 0.468–0.694). Rural residence was associated with lower adherence compared to urban residence (aCSHR = 0.616; CI: 0.516–0.735).

**Conclusion:**

Female sex and higher baseline CD4 count were associated with increased risk of defaulting, while older age predicted mortality following CD4 recovery. Adherence was associated with female sex, urban residence, tuberculosis co-infection, and viral load. Mortality estimates were limited by a few events, leading to imprecision in some covariates. Targeted retention strategies, strengthened rural health services, and monitoring of older patients are essential to sustain long-term treatment outcomes after immunological recovery.

## Introduction

Adherence to combination ART is essential for improving survival, reducing morbidity, and quality of life among people living with HIV PLWH) (1–4). Adherence to ART reduces the likelihood of transmitting HIV to uninfected partners, slowing disease progression and immunological recovery through viral suppression (5–7). Antiretroviral therapy (ART) is a lifelong treatment that requires sustained adherence to maintain viral suppression, preserve immune function, and prevent disease progression (8, 9). However, treatment interruptions may occur due to various clinical, structural, and socioeconomic circumstances, including access barriers, adverse drug effects, and health system challenges, potentially increasing the risk of opportunistic infections.

Treatment interruptions may be planned or unplanned, short-term or long-term, or permanent. The patient or service provider may choose to discontinue ART for various reasons. The patient factors may include lack of self-motivation for taking medication, lack of family support, workplace discrimination through disclosure of HIV status, religious beliefs, switching of locations, and poor patient-provider relationships (10–12). Some of the reasons a provider may recommend stopping treatment include severe drug poisoning, intermediate-stage disease, surgery that precludes oral treatment, or the absence of antiviral medication (13). Discontinuation of ART leads to suboptimal clinical outcomes, viral resistance, diminished quality of life, higher risk of opportunistic complications, loss of income and mortality (14, 15).

Several authors studied CD4 count recovery in the presence of competing events. For example (16), estimated the cumulative incidence of CD4 recovery of at least 500 cells/mm^3^ and identified associated factors, whilst considering virological failure, loss to follow-up, and mortality as competing events. The results showed that patients with higher baseline CD4 counts were a predictor of CD4 recovery (17). focused on the competing events of CD4 count recovery to the development of a serious AIDS event, a serious non-AIDS event (SNAE), or mortality from any cause (AIDS or non-AIDS or of unknown cause). They concluded that patients who initiated ART with a CD4 count of less than 200 cells/mm^3^ remained at risk after CD4 count recovery. Comparison of mortality, loss to follow-up (LTFU), and immunological and virological outcomes among patients who spent 5 years on ART. Older adults had better virological outcomes, minimised LTFU, higher mortality and slower immunological recovery (18).

Maintaining a CD4 count above 500 cells/mm3 is the ultimate goal for patients taking ARVs To achieve the best possible response and lessen the likelihood of transmission of the disease and HIV-related mortality, long-term adherence to ART is essential (19). Taking antiretroviral consistently keeps HIV from increasing, which diminishes the risk of immunological failure. After the attainment of CD4 count recovery, some patients might continue adhering to the ART, others may stop taking medication for various reasons, while others may die because of medication resistance, treatment failure or opportunistic infections (20–22). For HIV control, it is important to know the factors associated with these three events. Therefore, this study uses a competing risk regression model to determine the contributors to mortality, defaulting, and adherence to ART as competing events among CD4 count-recovered patients on ART.

## Methods

### Study setting

The study was conducted in eThekwini (urban) and Vulindlela (rural) in KwaZulu-Natal, South Africa, a province with one of the highest HIV prevalence rates in the country. These sites were selected to reflect contrasting healthcare contexts within a high-burden setting. eThekwini is a densely populated metropolitan area with greater access to specialised HIV services, whereas Vulindlela is a rural area characterised by limited healthcare infrastructure, higher poverty levels, and longer travel distances to clinics. These settings allowed assessment of contextual influences on CD4 count recovery among patients initiating ART with advanced disease.

### Study design

A retrospective cohort study was conducted among HIV-infected patients initiating ART at the research institution, South African Centre for the AIDS Program of Research (CAPRISA).

### Study population

This study used data from the Centre for the AIDS Programme of Research in South Africa (CAPRISA), an institute which focuses on HIV/AIDS and Tuberculosis (TB) research, particularly in high-burden communities. The program enrolled 4,013 HIV patients for antiretroviral therapy initiation between June 2004 and August 2013.

### Inclusion and exclusion criteria

Patients who met the 2004 eligibility criteria of ART initiation with a CD4 count below 200 cells/mm^3^ or WHO stage 4 AIDS-defining illness were 2,528 (23). Of these patients, 726 reached CD4 recovery of ≥500 cells/mm^3^ and were included in this analysis. Patients whose CD4 counts were ≥200 at baseline were excluded in the study.

### Bias

The study used a complete case analysis to avoid bias.

### Variables

CD4 count, viral load, demographic characteristics (location, age, gender), Tuberculosis status, WHO stages and medication regimens were recorded at baseline. The standard first-line ART was two nucleoside reverse transcriptase inhibitors and a non-nucleoside reverse transcriptase inhibitor. Second-line treatment comprises a protease inhibitor (PI) plus two nucleoside analogues (NRTIs) and is suitable for patients who have failed first-line treatment. CD4 counts and viral load measurements were obtained at baseline, every 6 months, or as needed for clinical reasons. Clinic attendance records were used to define adherence. Patients were considered adherent if they consistently attended scheduled ART follow-up appointments without missed visits. Defaulting was defined as absence from the clinic and loss to follow-up. Time to death was defined as the interval from ART initiation to the recorded date of death in the cohort database. Participants who remained alive at the end of the study were censored.

### Statistical methods

Patients' characteristics were summarised using descriptive statistics including median and interquartile range for continuous covariates and frequencies for categorical measures. The Fisher's Exact tests were used to find the relationship among categorical variables. The chi-squared was not appropriate as its assumptions were violated in some categories. The *t*-test and Mann–Whitney test were used to explore associations between continuous variables, with *p*-values less than 0.2 indicating statistical significance. All the association tests used were two-sided tests.

The competing events among CD4-count-recovered patients after ART initiation were mortality, defaulting, and adherence. The follow-up time was from the time the patient attained CD4 count recovery until they experienced any competing event. Adherence was handled as a programmatic endpoint and modelled as a competing event. This strategy allows for direct estimation of cause-specific hazards for the different treatment trajectories. The cumulative incidence function was used to assess the effects of covariates: gender, tuberculosis, site or location, and viral load. We performed bivariate cause-specific competing-risk analyses of baseline characteristics for the three competing events. Variables with a *p*-value < 0.2 were included in the multivariable cause-specific competing risks model. Tuberculosis and WHO stages had wide, overlapping confidence intervals due to small cell counts under the competing event “mortality”. All statistical analysis was performed by the R Comprisk package.

### Statistical formulation of cause cause-specific hazard model

Let T and C represent the failure time and censoring time, respectively. In datasets with K competing risks, we observe the pair (X, *δ*), where X = min(T, C) and *δ* = 1,…,K serves as an indicator, taking the value of 0 for censoring and other values to identify specific causes of failure. For analysing competing-risk data, two important metrics are the cause-specific hazard function and the cumulative incidence function. The cause-specific hazard function $h_{k}(t)$ (Equation 1) At time *t*, it indicates the instantaneous failure rate due to cause *k*, given survival until time *t* or beyond.

It is defined as:

$$h_{k}(t)=\underset{\Delta t\overset{}{\to }0}{\lim}\frac{P(t<T<T+\Delta t,\delta =k|T>t)}{\Delta t}\;\mathrm{for}\;k=1,\ldots ,K$$ (1)

The cumulative incidence function, denoted by $F_{k}(t)$ (Equation 2) is the probability of failure due to cause *k* prior to time *t* and is given by:

$$F_{k}(t)=P(T\leq t,\delta =k)\;\mathrm{where}\;k=1,\ldots ,K.$$ (2)

The cumulative incidence function is also referred to as the subdistribution function, because it is not a true probability distribution. The $k^{th}$ (Equation 3) cumulative incidence function can be expressed mathematically in terms of all the cause-specific hazard functions via the integral as follows,

$$F_{k}(t)=\int_{0}^{t}S(u)h_{k}(u)du=\int_{0}^{t}S(u)dH_{k}(u)\;\mathrm{for}\;k=1,\ldots ,K\;$$ (3)

Where $H_{k}(t)=\int_{0}^{t}h_{k}(u)du$ is the cause-specific cumulative hazard function and $S(t)=exp\left(-\sum_{k=1}^{K}H_{k}(t)\right)$ is the overall survival function, which is the probability of surviving beyond time *t*. It is clear that $F_{k}(t)$ involves not only $h_{k}(t)$ but also all the competing cause-specific hazard functions when K>1. When K=1, the subdistribution function degenerates to $F_{1}(t)=1-exp(-H_{1}(t))$ and becomes a function of only $h_{1}(t)$. In the presence of competing risks, the cumulative incidence of a specific event depends not only on its own hazard rate but also on the hazard rates of all other competing events.

### Ethics approval and consent to participate

This study used secondary data from CAPRISA, which received ethical approval from the University of KwaZulu-Natal Biomedical Research Ethics Committee (Ref: E248/05), in accordance with regulations governing the protection of human subjects. Informed consent was obtained from each participant or their guardian (if the participant was under 18 years old) at the time of ART initiation. Additionally, this study does not include any identifying information about the participants; thus, consent for publication is not applicable.

### Availability of data and materials

CAPRISA has a defined procedure to facilitate broader access to its research data. Details regarding how to request and obtain data can be found on the CAPRISA website. Any investigator can request data sets used in analyses for published CAPRISA research articles by submitting an online request to the CAPRISA Scientific Review Committee. Once approved, the requested data sets, study protocol, and statistical analysis plan will be provided to the investigating party.

## Results

### Participants

Table 1 presents the demographic and clinical characteristics of the 726 HIV patients who recovered their CD4 count, after initiating ART with CD4 < 200 cells/mm^3^.

**Table 1:** Demographic and clinical characteristics for CD4 count recovered patients between June 2004 and August 2013 in KwaZulu-Natal, South Africa.

Characteristics	Defaulted (48) (7%)	Mortality (15) (2%)	Adherence (663) (91%)	*p*-value
**The site**, *n* (%)				
Urban (eThekwini)	4 (1%)	1 (0%)	165 (23%)	0.007
Rural (Vulindlela)	44 (6%)	14 (1%)	498 (69%)	
Age*, median (IQR)	30 (25.75–36)	39 (32–49.75)	32 (28–38)	<0.001
WHO Stage** *n*(%)				
1	12 (2%)	2 (0%)	12 (14%)	
2	9 (1%)	4 (1%)	17 (19%)	0.183
3	23 (3%)	8 (1%)	54 (59%)	
4	3 (1%)	1 (0%)	8 (9%)	
**TB status**, *n* (%)				
TB present	6 (1%)	0(%)	127 (17%)	0.090
No TB	42 (6%)	15 (2%)	536 (74%)	
**Viral load** (copies/mL)				
>400	14 (2%)	3 (1%)	96 (13%)	0.022
≤400	34 (4%)	12 (2%)	567 (78%)	
**Regimen**, *n*				
Regimen 1 (First line)	37 (5%)	9 (1%)	539 (74%)	0.086
Regimen 2 (Second line)	11 (2%)	6 (1%)	124 (17%)	
**Gender**, *n*(%)				
Female	32 (4%)	9 (1%)	501 (69%)	0.138
Male	16 (3%)	6 (1%)	162 (22%)	
**CD4count** median (IQR)	126.5 (85–161.25)	122 (61.5–145)	108 (51.5–153)	<0.001

* 2 missing age.

** 2 missing WHO stages.

### Exploratory data analysis

Among patients who recovered their CD4 count 48(7%) defaulted, 15(2%) died and 663(91%) were adherent. Females who defaulted were 32(4%), 9(1%) died and 501(69%) were adherent. The median and inter-quartile range for patients' age who defaulted were 30(25.75–36), 39(32–49.75) died, and 32(28–38) were adherent. WHO stage 3 recorded 23(3%) defaulters, 8(1%) mortality and 338(54%) were adherent. Patients without tuberculosis were adherent to treatment 536(74%), and 42(6%) defaulted. Rural patients who died were 14(1%), 44(6%) defaulted and 498(69%) adhered to treatment. The median and inter-quartile range CD4 count for patients who defaulted were 126.5(85–161.25), 122(61.5–145) died and 108(51.5–153) adhered to treatment. Patients who died on first-line treatment were 9(1%), 539(74%) adhered to treatment and 37(5%) defaulted. Patients who died with a viral load of less than 400 copies/mL were 12(2%), 34(4%) defaulted, and 567(78%) adhered to treatment.

### Cumulative incidence functions analyses

**Figure 1:**
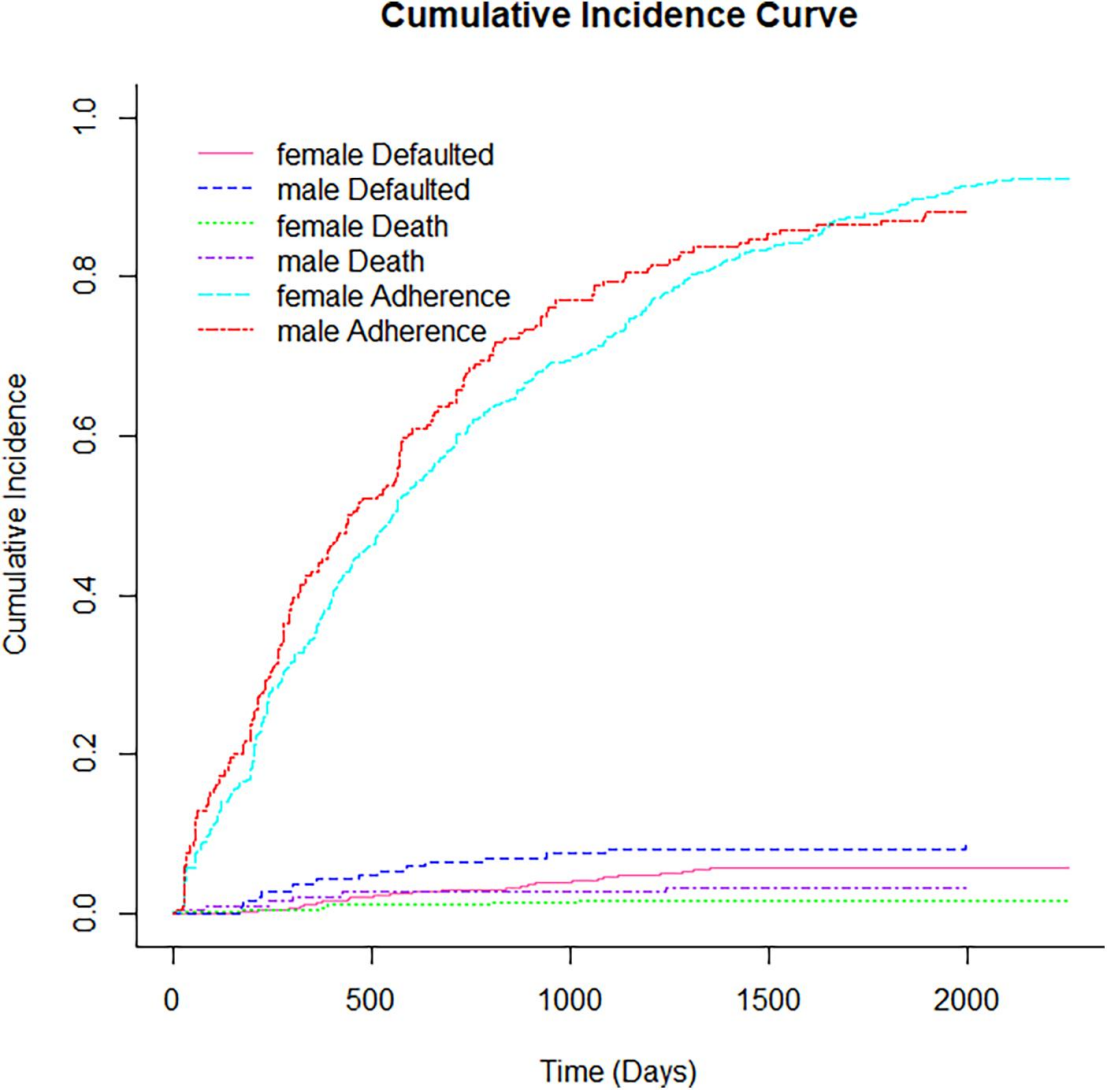
Cumulative incidence function for gender.

**Figure 2:**
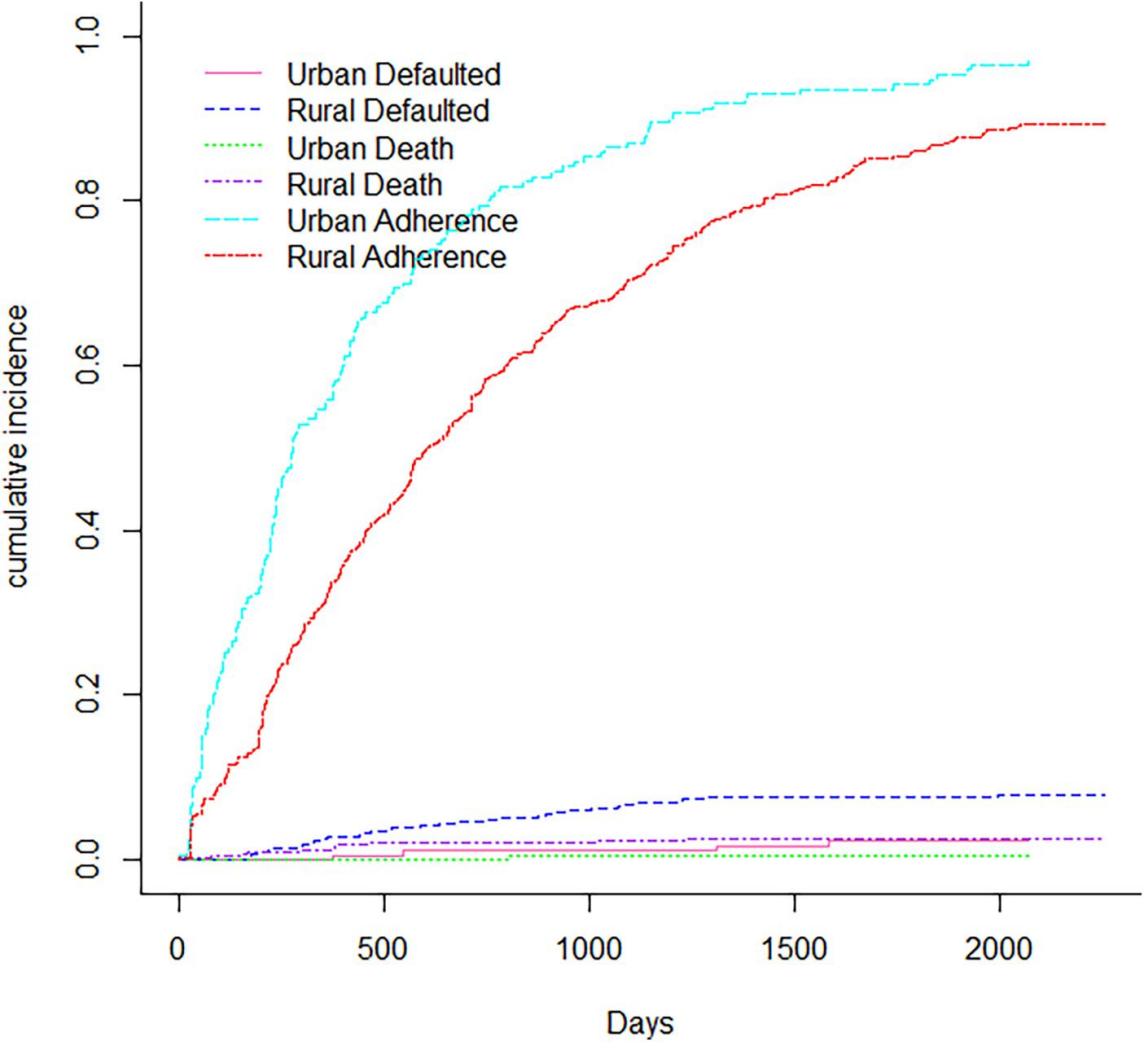
Cumulative incidence function for site.

**Figure 3:**
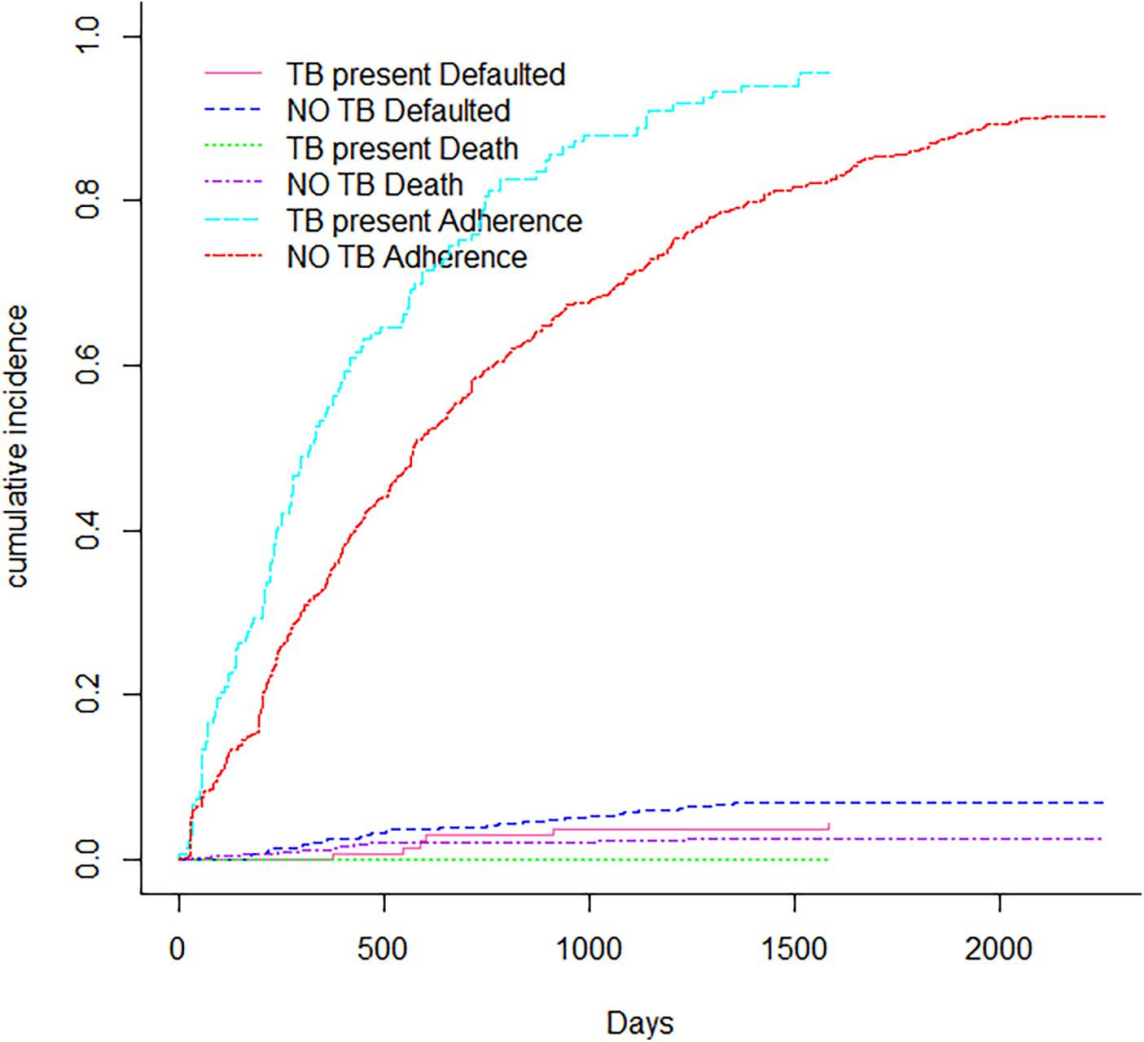
Cumulative incidence function for tuberculosis.

**Figure 4:**
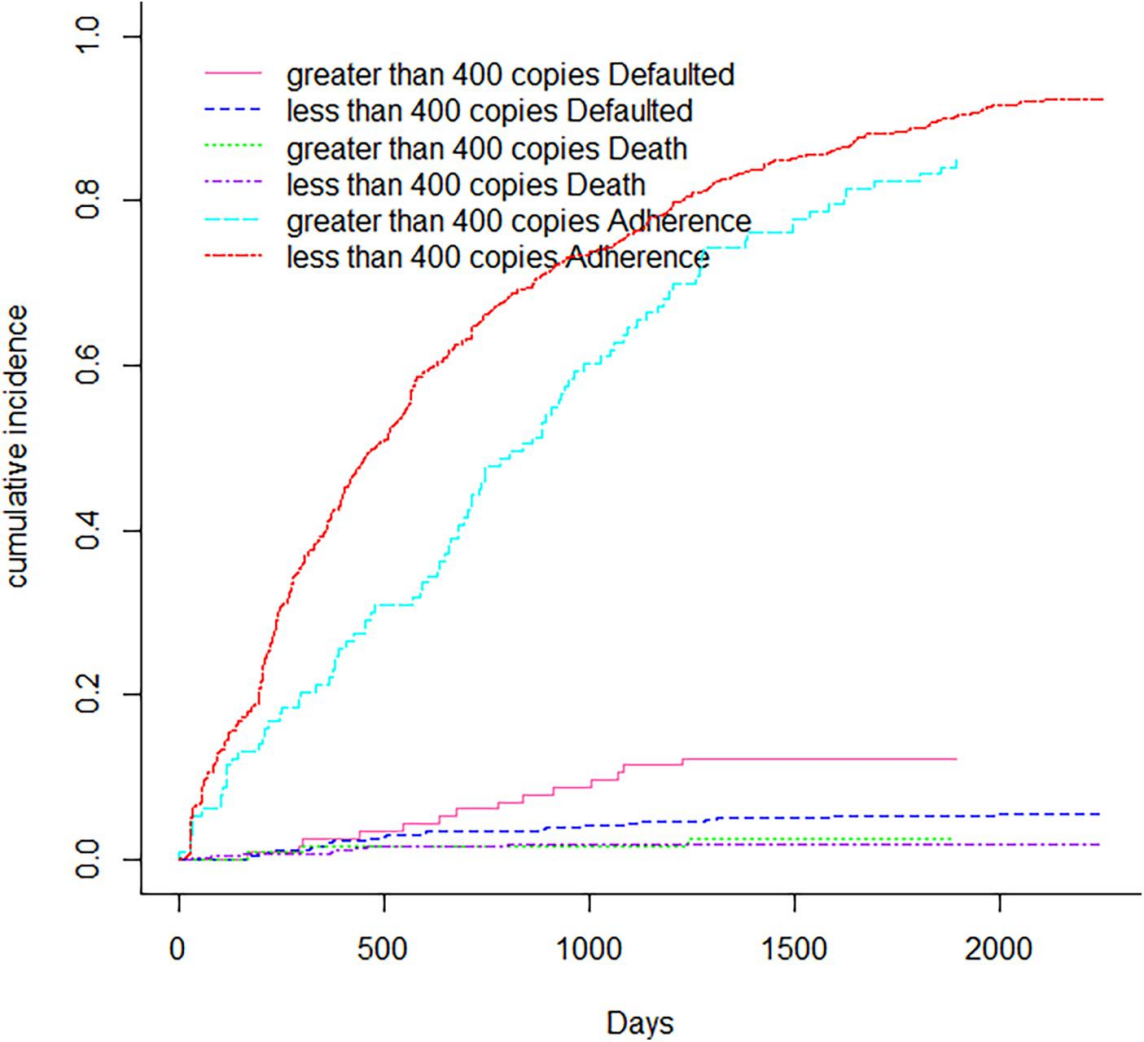
Cumulative incidence function for viral load.

Figure 1 displays non-parametric estimates of the cumulative incidence functions for mortality, default and adherence as competing risks. It showed that males had a higher risk of adherence, defaulting and mortality than females. Figure 2 showed that the Cumulative incidence for rural patients was at a higher risk of defaulting and dying than urban patients, while urban patients had a higher risk of adherence than rural patients did. The prevalence of tuberculosis patients had a risk of adherence, while those who had no tuberculosis had a higher risk of defaulting and mortality, as shown in Figure 3. Figure 4 showed that patients with viral load of at most 400 copies/mL had a higher risk of adherence, whereas those with viral load above 400 copies/mL had a significantly higher risk of default and mortality.

### Competing risk regression analysis

Table 2 presents the results of a bivariate competing risk regression analysis examining predictors of mortality, defaulting, and adherence, treating these outcomes as competing events. Viral load, gender, and CD4 count were significant predictors of defaulting. Females had a significantly higher risk of defaulting compared to males [adjusted cause-specific hazard ratio (aCSHR) = 2.161, 95% confidence interval (CI): 1.182–3.958, *p* = 0.013], indicating that after adjusting for other covariates, females were approximately 2.16 times more likely to default from care. Higher CD4 count was associated with a marginally greater hazard of defaulting [aCSHR = 1.005, 95% CI: 1.000–1.010, *p* = 0.042], possibly reflecting that individuals with improved immunological status may perceive themselves as healthier and become less engaged in care. Patients with viral load >400 copies/mL exhibited a lower hazard of defaulting, though this finding was marginally non-significant (aCSHR = 0.570, *p* = 0.079); this trend may be attributable to closer clinical monitoring among those with unsuppressed viral load.

**Table 2:** Bivariate competing risk regression analysis for predictors of mortality, defaulting and adherence among CD4 count recovered patients in KwaZulu-Natal, South Africa between June 2004 and August 2013.

	Defaulted		Mortality		Adherence	
Characteristics	aCSHR (95% CI)	*p*-value	aCSHR (95% CI)	*p*-value	aCSHR (95% CI)	*p*-value
Gender (Ref: Male)	2.161 (1.182–3.958)	0.013*	2.426 (0.859–6.851)	0.094	1.314 (1.099–1.571)	0.003*
Female						
Age	0.981 (0.947–1.017)	0.293	1.091 (1.036–1.149)	0.001	1.005 (0.996–1.014)	0.312
TB (Ref: TB present)	0.887 (0.374–2.104)	0.786	84,86,6737 (0-∞)	0.998	0.570 (0.468–0.694)	<0.001*
NO TB						
WHO stage1	0.371 (0.048–2.892)	0.344	11,06,8759 (0-∞)	0.998	3.77 (0.523–27.16)	0.188
WHO stage2	0.228 (0.029–1.821)	0.163	17,01,475 (0-∞)	0.998	4.364 (0.607–31.37)	0.143
WHO stage3	0.193 (0.026–1.445)	0.109	11,36,267 (0-∞)	0.998.	4.336 (0.607–30.97)	0.144
WHO stage4 (Ref)						
Site (Ref: Urban)	2.077 (0.745–5.786)	0.162*	3.042 (0.399–23.19)	0.283	0.616 (0.516–0.735)	<0.001*
Rural						
Regimen (Ref: First line treatment)	0.802 (0.408–1.577)	0.522	2.133 (0.754–6.03)	0.153	0.648 (0.532–0.789)	<0.001*
Second line treatment						
Viral load (Ref: ≤400 copies/mL)	0.570 (0.308–1.068)	0.079*	0.912 (0.257–3.237)	0.886	1.319 (1.061–1.639)	0.013*
>400 copies/mL						
CD4 count	1.005 (1–1.01)	0.042*	1.001 (0.992–1.009)	0.868	0.999(0.998–1.001)	0.913

* Means significant variables.

Age emerged as the sole significant predictor of mortality (aCSHR = 1.091, 95% CI: 1.036–1.149, *p* = 0.001), with each one-year increase in age associated with a 9.1% increase in the hazard of mortality. This suggests that older patients remain vulnerable despite immunological recovery. Gender, TB status, WHO stage, site, treatment regimen, viral load, and CD4 count were not significantly associated with mortality. The extremely large hazard ratios accompanied by infinite confidence intervals for TB status and WHO stage reflect sparse mortality events in those categories, yielding unstable estimates.

Significant predictors of adherence included gender, TB status, site, treatment regimen, and viral load. Females had a significantly higher likelihood of adherence than males (aCSHR = 1.314, 95% CI: 1.099–1.571, *p* = 0.003), corresponding to an approximate 31% probability of achieving adherence. Patients without TB had a clinical follow-up and monitoring among co-infected individuals. Patients attending rural facilities demonstrated a significantly lower hazard of adherence relative to urban patients (aCSHR = 0.616, 95% CI: 0.516–0.735, *p* < 0.001), likely reflecting structural barriers such as limited healthcare access, transportation challenges, and resource constraints in rural settings. Age, WHO stage, and CD4 count were not significantly associated with adherence.

### Penalized Cox regression model (firth correction)

Sparse mortality events were addressed using a penalised Cox regression with Firth's correction (Table 3). In the primary cause-specific hazard analysis, estimates for tuberculosis status and WHO stage were highly unstable in the standard model, yielding extremely large hazard ratios and non-estimable confidence intervals indicative of quasi-complete separation. The Firth penalised model produced finite, more stable estimates, though several covariate confidence intervals remained wide, reflecting the small number of events. The association between increasing age and mortality remained statistically significant and consistent across models. Estimates of tuberculosis status and WHO stage, while no longer infinite, remained imprecise. In summary, the penalised approach mitigated numerical instability and separation but could not fully overcome the limitations imposed by sparse deaths; mortality-related estimates should therefore be interpreted with caution.

**Table 3:** Penalized Cox regression model (firth correction).

Characteristics	HR (95% CI)	*p*-value
Gender (Ref: Male)	3.108 (1.006–9.299)	0.049
Female		
TB (Ref: TB present)	7.623 (0.732–1,041.158)	0.098
NO TB		
Site (Ref: Urban)	3.192 (0.652–31.254	0.164
Rural		
Regimen (Ref: First line treatment)	5.774 (1.832–17.250	0.004
Second line treatment		
Viral load (Ref: ≤400 copies/mL)	0.916 (0.276–3.814)	0.893
>400 copies/mL		
CD4 count	0.998 (0.989–1.832)	0.681
Age	1.102 (1.047–1.159)	<0.001

## Discussion

This study followed HIV patients who had attained immunological recovery (CD4 ≥ 500 cells/mm³) after ART initiation and evaluated the occurrence of competing clinical outcomes, namely mortality, treatment default, and sustained adherence. The competing risks framework was appropriate because these outcomes are mutually exclusive and represent alternative clinical trajectories following immune recovery. Similar cohort-based competing risks analyses in African HIV populations have demonstrated that mortality, loss to follow-up, and treatment adherence represent distinct but interrelated pathways influencing long-term treatment success and survival (24).

Baseline CD4 count was significantly associated with treatment default in the study, indicating that HIV patients with higher CD4 counts had an elevated hazard of defaulting on ART. This finding may reflect the perception of improved health among HIV patients who experience immunological recovery, leading to lessened engagement in long-term treatment programs. Previous studies have consistently shown that CD4 count at ART initiation is a strong predictor of disease progression and mortality, with lower CD4 levels associated with higher morbidity and mortality risk (25, 26). Disengagement among clinically stable individuals has been increasingly recognised in the treat-all era (27). These findings suggest that retention strategies should not be limited to clinically unstable patients. Adherence reinforcement at the point of immunological recovery, multi-month dispensing (MMD), and differentiated service delivery (DSD) models that stratify patients by engagement risk have demonstrated improved retention in sub-Saharan Africa (28, 29).

Females had a significantly higher hazard of defaulting but were also more likely to maintain sustained adherence from care than males. Although females exhibited a higher mortality risk, this difference was not statistically significant. These results highlight the significance of gender-responsive retention tactics, including differentiated care tailored to women. Furthermore, community-based ART delivery, flexible clinic hours, integration of HIV care with sexual and reproductive health services, and gender-transformative interventions that address stigma and economic vulnerability are examples of practical treatments (30, 31). Recent research in sub-Saharan Africa demonstrates that gender-sensitive, community-centred service delivery improves retention and viral suppression outcomes (32).

Age was significantly associated with mortality, with older HIV patients experiencing a higher hazard of mortality. Ageing populations living with HIV face multi-morbidity, frailty, and incomplete immune recovery despite viral suppression (33, 34). WHO and regional HIV guidance now emphasise HIV and non-communicable disease (NCD) services, routine cardiovascular and metabolic screening, and age-adapted monitoring strategies (35). These findings reinforce the need for integrated, life-course HIV care models rather than uniform treatment protocols.

Geographic location was another important determinant of treatment outcomes. HIV patients residing in rural areas had a significantly lower hazard of adherence compared with urban HIV patients. Transport costs, workforce shortages, and limited infrastructure remain major determinants of rural disengagement (36, 37). Evidence from other studies showed that community ART groups, task-shifting to community health workers, mobile outreach clinics, and multi-month ART dispensing significantly improve retention while optimizing constrained health system resources (29, 38). These interventions are particularly relevant in rural settings where health workforce density is limited. Furthermore, population-based studies have shown that residence in resource-limited settings is associated with poorer viral suppression and greater risk of treatment failure (39).

Viral load was significantly associated with adherence outcomes, with higher viral loads HIV patients experiencing an elevated hazard of adherence events. This likely reflects intensified adherence counselling and closer follow-up for HIV patients with detectable viral load to improve re-suppression rates (40). Viral load is a key indicator of treatment effectiveness and reflects the level of viral suppression achieved through ART adherence (41, 42). Monitoring viral load is essential for evaluating treatment response and identifying HIV patients at risk of treatment failure. Studies across sub-Saharan Africa have emphasised the importance of viral load suppression as a critical marker of treatment success and long-term survival of HIV patients (42).

Treatment regimen type was also associated with adherence outcomes, with HIV patients on second-line therapy demonstrating a lower hazard of adherence than those on first-line regimens. Transition to second-line regimens often follows prior treatment failure and may involve increased pill burden or adverse effects (22, 39). Studies conducted between 2020 and 2024 indicate that enhanced counselling at regimen switch, early follow-up visits, and peer-support interventions improve adherence durability among treatment-experienced patients (43).

## Limitations

The cohort was initiated during an early phase of ART program expansion in South Africa, when routine clinical documentation prioritised treatment line rather than detailed drug-level combinations, and systematic recording of comorbidities beyond tuberculosis was limited. The analyses focused solely on first- vs. second-line therapy and on tuberculosis status, the only opportunistic infection consistently recorded. Changes in ART formulations, toxicity profiles, and supportive care strategies over time may have introduced residual confounding. Therefore, results should be understood in the historical context of ART delivery before the advent of modern integrase inhibitor regimens and enhanced comorbidity monitoring. The limited number of mortality cases also raises concerns regarding potential overfitting and small-sample bias in regression modelling. When events per variable are low, hazard ratio estimates may become unstable and confidence intervals excessively wide. Future studies with larger sample sizes would provide a more robust estimation of mortality predictors among HIV patients with advanced HIV disease.

## Conclusion

Significant variables associated with adherence in the presence of other competing events were female gender, tuberculosis, location, regimen and viral load. Contributors for defaulting were gender and baseline CD4 count, whilst age was associated with mortality. These findings highlight the need for women-focused differentiated strategies, strengthened adherence support for clinically stable HIV patients, and integrated HIV-non communicable diseases (NCD) for older adults. Community ART groups, decentralised ART distribution and multi-month dispensing strategies should be implemented to account for existing structural and resource constraints in rural health systems. Implement enhanced adherence counselling at regimen switch for HIV patients transitioning to second-line regimens. Larger, prospective studies with extended follow-up are required to elucidate determinants of mortality and to assess the impact of evolving ART strategies in HIV patients presenting with advanced HIV disease.

### What is known about this topic

- Adherence to ART reduces the likelihood of transmitting HIV to uninfected partners, slowing disease progression and immunological recovery through viral suppression.
- Treatment interruptions may cause poor quality of life in patients and in other cases, HIV-related mortality.
- Patients initiating ART with low CD4 count remain at risk after CD4 count recovery.

### What this study adds

- For HIV control, factors associated with CD4 count recovery are tuberculosis, location, regimen and viral load.
- Improved health facilities in rural areas and close monitoring of patients without tuberculosis are recommended.
- Significant contributors to defaulting are gender and baseline CD4 count.

## Data Availability

The data analyzed in this study is subject to the following licenses/restrictions: Data is available upon request from CAPRISA. Requests to access these datasets should be directed to batidzirai@ukzn.ac.za.
